# High effects of climate oscillations on population diversity and structure of endangered *Myricaria laxiflora*

**DOI:** 10.3389/fpls.2024.1338711

**Published:** 2024-02-28

**Authors:** Hao Li, Guiyun Huang, Liwen Qiu, Jihong Liu, Yinhua Guo

**Affiliations:** ^1^ Yangtze River Biodiversity Research Center, Yangtze Eco-Environment Engineering Research Center, China Three Gorges Corporation, Wuhan, China; ^2^ National Engineering Research Center of Eco-Environment Protection for Yangtze River Economic Belt, Beijing, China; ^3^ College of Horticulture and Forestry, Huazhong Agricultural University, Wuhan, China

**Keywords:** *Myricaria laxiflora*, population structure, flooding stress, climate oscillations, three gorges reservoir, conservation

## Abstract

Exploring the effects of climate oscillations on the population diversity and structure of endangered organisms in the Three Gorges Reservoir (TGR) area is essential for hydrological environment changes on endangered organism evolution. *Myricaria laxiflora* is an endemic and endangered shrub restricted to the TGR along the banks of Yangtze River, China. Recently, six natural populations of this species were newly found upstream and downstream of the TGR, whose habitats have been dramatically changed by the summer flooding regulated by large dams. To study the water level fluctuations and climatic shifts on the genetic diversity and genetic differentiation of the six natural populations, 303 individuals from six populations were analyzed based on one nuclear DNA (ITS) and four chloroplast fragments (*trnL-F*, *psbA-trnH*, *rps16*, and *rpl16*). The phylogenetic tree and significant genetic divergence identified in the cpDNA and ITS with genetic isolation and limited gene flow among regions suggested that the six populations separated well to two groups distributed upstream and downstream. The MaxEnt modeling results indicated that obvious unidirectional eastward migration via Yangtze River gorges watercourse mediated from Last Interglacial to Last Glacial Maximum were showed with the narrow scale distributions of six remnant populations and nine extirpated populations. The initial habitat fragmentation could be triggered by the accumulation of local habitat loss of the impoundment of the TGR during the Present period and might remain stable restoration with bidirectional diffusion in the Future. Divergences among *M*. *laxiflora* populations might have been induced by the drastic changes of the external environment and limited seed/pollen dispersal capacity, as the results of long-term ecological adaptability of summer flooding stress. The haplotypes of nuclear gene could be used for population’s differentiation and germplasm protection. This identified gene flow and range dynamics have provided support for the gene-flow and geology hypothesis. It is also crucial for rescuing conservation to understand the impact of environmental dynamics on endangered organism evolution.

## Introduction

*Myricaria laxiflora*, an endemic and endangered aquatic shrub, is restricted to the low-altitude riverbank of the Three Gorges area along the banks of Yangtze River, China. It is the only exception in genus *Myricaria* (Tamaricaceae), the majority of which occur in high altitudes above 1000 m (Zhang and Zhang, 1984). Since the middle 1990s, tremendous efforts on conservation of this species have been undertaken (Wang et al., 2003). The construction and operation of the world’s largest cascade water conservancy and hydropower projects (Xiangjia Dam, Three Gorges Dam, and Gezhou Dam) has dramatically changed upstream and downstream water-level and climatic fluctuations of the Three Gorges Reservoir (TGR) area for power generation and flood control, which leads to flooding of most habitats of this species, making it a national grade II endangered species (Chen et al., 2005). *M. laxiflora* experiences prolonged flooding stress from June to September each year, so the summer flooding used to be considered as an ecological process that affected the development and evolution of this species (Chen and Xie, 2007). All the natural populations of *M. laxiflora* in the TGR have been destroyed by the impoundment, and the newly found six remnant populations have been also severely affected by the summer flooding (Wu et al., 1998; Wang et al., 2003; Liu et al., 2006). It was considered to be narrowly distributed in the low-altitude riverbank of 70 m to 155 m (Zhang and Zhang, 1984), but three remnant populations of *M. laxiflora* were newly found upstream of the TGR in Yibin, and three natural populations found downstream in Yichang (Huang et al., 2023). The Zhijiang population (ZJ) occurs at a location furthest downstream with a relatively low altitude of 42 m, and the Yibin population (YB) occurs at a location furthest upstream with a relatively high altitude of 263 m (**Table 1**). The entire natural distribution range of *M. laxiflora* is greatly expanded by the recently discovered remnant populations, which is essential significance for the conservation and recovery.

**Table 1:** Sampling sites within *M. laxiflora* populations from the Yangtze River valley.

Species name	Population site	Longitude	Latitude	Altitude (m)
	Within the TGR (extirpated population group)			
*M. laxiflora*	Zitong, Banan (ZT)	106.977	29.769	154
*M. laxiflora*	Shangzhongba, Fuling	107.165	29.695	147
*M. laxiflora*	Heping, Fengdu	107.741	29.907	144
*M. laxiflora*	Tangtuba, Zhongxian	107.975	30.206	140
*M. laxiflora*	Ganjing, Zhongxian	108.109	30.296	136
*M. laxiflora*	Huangshizui, Fengjie	109.254	30.956	113
*M. laxiflora*	Xiuqiubao, Wushan	109.769	31.042	95
*M. laxiflora*	Wangjiang, Zigui (WJ)	110.68	31.004	78
*M. laxiflora*	Lanlingxi, Zigui	110.932	30.873	75
	Upstream the TGR (remnant population group)			
*M. laxiflora*	Yibin,Sichuan (YB)	105.035	28.776	263
*M. laxiflora*	Xuzhou, Yibin (XZ)	104.283	28.889	260
*M. laxiflora*	Luzhou, Sichuan(LZ)	105.226	28.756	258
	Downstream the TGR (remnant population group)			
*M. laxiflora*	Yanzhiba,Yichang (YZ)	111.324	30.649	46
*M. laxiflora*	Guanzhoudao, Yidu (YD)	111.566	30.253	44
*M. laxiflora*	Dongshi, Zhijiang (ZJ)	111.696	30.398	42
	Introduced individuals			
*M. laxiflora*	Beijing Botany (BJ)	–	–	–
*M. laxiflora*	Wuhan Botany (WH)	–	–	–

The Three Gorges area is a biodiversity hotspot in central China, with many riparian plants highly susceptible to environmental change. Over a long period of evolution, these aquatic plants have adapted to the natural climate dynamics of seasonal fluctuations (Poff et al., 1997; Stromberg, 2001; Imbert and Lefèvre, 2003). The genetic diversity of distribution range may be directly reduced by the dramatic environment, which will extremely impact on the adaptability and sustainability of riparian plants. Therefore, habitat loss and fragmentation could increase the risk of decline and extinction on aquatic plants in the TGR (Wu et al., 2003; Liu et al., 2006). The diversity persistence of the riparian plant species and populations is determined by the long-term seasonal fluctuations of unregulated water level, flood disturbance, and other climate factors (Stromberg, 2001; Johnson, 2002). The impacts on riparian plants in new habitat of regulated summer flooding have never been researched with such dramatic hydrology changes.

*M. laxiflora* with strong tolerance is seriously stressed by summer flooding and water submergence with a reversed lifestyle of summer dormancy and winter growth (Liu et al., 2006; Chen and Xie, 2007). It has mixed characteristics of sexual and asexual reproduction with excessive seed production and highly clonal propagation through efficient wind and water dispersal (Wu et al., 1998; Shen et al., 1999; Wang et al., 2003). The inter-simple sequence repeat (ISSR) data and amplified fragment length polymorphisms (AFLPs) of *M. laxiflora* has been used to detect high levels of genetic diversity within populations and species (Liu et al., 2006; Tian et al., 2012). The species-level phylogeographical patterns of *Myricaria* species in western China and the origin of *M. laxiflora* have been focused on only based on chloroplast haplotypes without nuclear DNA (ITS), which is incomplete fragments of Chongqing populations and only haplotypes of *psb A-trn H* and *rpL16* (Liu et al., 2009).The basic goal of ecological genetic studies is in its full natural range to understand the population diversity and structure of a species (Jørgensen and Mauricio, 2004). Previous research had limited value in determining conservation strategies, because several recently discovered upstream and downstream populations were not all included (Li et al., 2003; Wang et al., 2003). In this study, the nuclear gene and chloroplast genes were identified based on materials from six remnant populations upstream and downstream of the TGR to study the population diversity and genetic structure of *Myricaria laxiflora*. The nine extirpated populations were also used to determine the mechanism of origin, divergence and population evolutionary history. The primary purpose of the present study was to (1) repartition the genetic diversity of *M. laxiflora* populations based on its entire natural distribution, (2) explore the origin and populations diversification throughout the TGR region and its adjacent areas, (3) explore the summer flooding and climatic changes that influenced natural distribution shifts and population differentiation of *Myricaria laxiflora*, and (4) assess the genetic integrity of the rescuing conservation. This valuable information is greatly contributed to formulate better strategies for conservation and reintroduction of *M. laxiflora* in the future.

## Materials and methods

### Sampling and flooding

A total of 303 individuals divided into two groups were collected from six populations at Yangtze River islands above water level in Yibin and Yichang, China. Through the data analysis of Yibin and Yichang water level stations from 2014 to 2019 (**Supplementary Figure 1**), the natural distribution area of the plant was completely submerged from June to October every year, and the downstream populations of the TGR was flooded a month earlier than the upstream. Samples of the six natural remnant populations covered almost the entire distribution of endangered *M*. *laxiflora* (**Table 1**). Within each population, 20–45 individuals were randomly chosen at least 20 m apart. Fresh leaves were immediately dried in silica gel and brought back to the laboratory for DNA extraction. Voucher specimens were deposited at Yangtze Botany Herbarium, Yangtze River Rare Plant Research Institute, China Three Gorges Corporation.

### DNA extraction and sequencing

Total genomic DNA was extracted from about 0.5 g dried leaves using a modified cetyltrimethylammonium bromide protocol (Doyle and Doyle, 1987). Four chloroplast fragments (*trnL-F*, *psbA-trnH*, *rps16*, and *rpl16*) (Shaw et al., 2005) and ITS (ITS4-5.8s RNA-ITS5) (White et al., 1990; Zhang et al., 2014) were amplified and sequenced. The polymerase chain reactions (PCRs) were performed in a total volume of 30 µL with 3 µL plant total DNA, 1.5 µL forward primer, 1.5 µL reverse primer (**Supplementary Table 1**), and 15 µL volume 2×Taq MasterMix (Cwbio, Beijing, China). PCR amplification has been confirmed by Gel electrophoresis analysis and purified for sequencing (**Supplementary Figure 2**). All sequences were deposited in GenBank with accession numbers OK135371-OK135422, OK265112-OK265317, AY207486, EU240609 and EU240610, KJ808608 and KJ808609, KJ808639 and KJ808640, and KJ808623 and KJ808624.

All the DNA sequences were edited by SeqMan (DNAstar package; DNAStar Inc., Madison, WI, USA) to obtain consensus sequences. The program MEGA-X was used to align for subsequent manual adjustments (Kumar et al., 2008). Haplotypes were identified and distinguished using DNAsp version 5.1 (Librado and Rozas, 2009). Gaps were treated as missing data during the tree searches. The phylogeny reconstruction based on haplotype was performed using maximum likelihood with IQ-TREE 1.2.12 (Nguyen et al., 2015). In addition, PopART 1.7 (Polzin and Daneshmand, 2003) was used to construct the geographic distribution of haplotypes in the map of Yangtze River system.

### Population genetics and structure

The principal components analysis (PCA) has been performed on a set of genetic sequences using the “adegenet and ggplot2 packages” in R Studio. The genetic sequences were initially processed with the “fasta2genlight” function, and the resulting PCA scores were visualized in a scatter plot. The “geom_point” function has been used to plot the points, and labels with the sequence names were added using “geom_text.” Haplotype diversity (Hd) (Nei and Tajima, 1981) and nucleotide diversity (π/Pi) (Nei and Li, 1979) for each population were calculated using DNAsp 5.1 (Librado and Rozas, 2009) to verify the degrees and patterns of diversity. PERMUT (Pons and Petit, 1996) was used to access within-population diversity (Hs) and population differentiation indices (Fst, Gst, and Nst) (Grivet and Petit, 2002).

The analyses of molecular variance (AMOVAs) with 1,000 permutations were performed using ARLEQUIN version 3.5 (Excoffier and Lischer, 2010) to detect the genetic variation at three level hierarchy of among regions (upstream and downstream TGR). Multivariate analysis of the data such as cluster analysis and multi-dimensional scaling (MDS) were performed with PRIMER 5 software. Hierarchical clustering and MDS was based on nucleotide diversity (Pi) of all populations’ chloroplast fragments. The coefficient of Stress was used to measure the effectiveness of the two-dimensional point distribution map in MDS. The Stress < 0.05 indicates that the results are highly representative. The results are generally reliable with 0.05 ≤ Stress < 0.1 and have some explanatory significance with 0.1 ≤ Stress < 0.2 (Khalaf and Kochzius, 2002).

### Demographic history and species distribution modeling

Neutrality test (Fu’s Fs; Tajima’s D) (Tajima, 1989) and mismatch distribution analysis (Schneider and Excoffier, 1999) were conducted to test whether there was potential population expansion of *M*. *laxiflora* (Harpending, 1994). MaxEnt 3.3.3K (Phillips et al., 2006) was used to predict the distribution of *M*. *laxiflora* during four time periods: Last Interglacial (LIG), Last Glacial Maximum (LGM), and the Present and Future. A total of 15 distribution sites acquired from field investigations and online herbarium records were used in analyses. Nineteen bioclimatic environment variables (Hijmans et al., 2005) were downloaded from the WorldClim dataset at 30 s resolution to detect changes in distribution between the four time periods. The area under the “Receiver Operating Characteristic curve” (AUC) (Peterson et al., 2008; Elith and Leathwick, 2009) was also tested to observe the accuracy of each model prediction (Fielding and Bell, 1997).

## Results

### Genetic diversity

**Figure 1:**
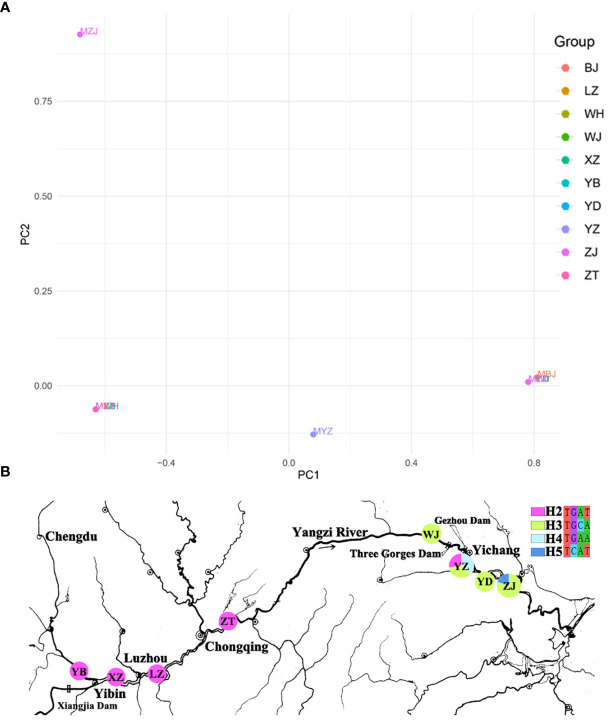
Genetic diversity and genetic differentiation of nuclear gene (ITS) for *M. laxiflora*. **(A)** The resulting PCA plot of nuclear gene (ITS). **(B)** The geographic distribution of nuclear gene (ITS). Each pie chart along the Yangzi River represents a population and each color represents a haplotype.

**Figure 2:**
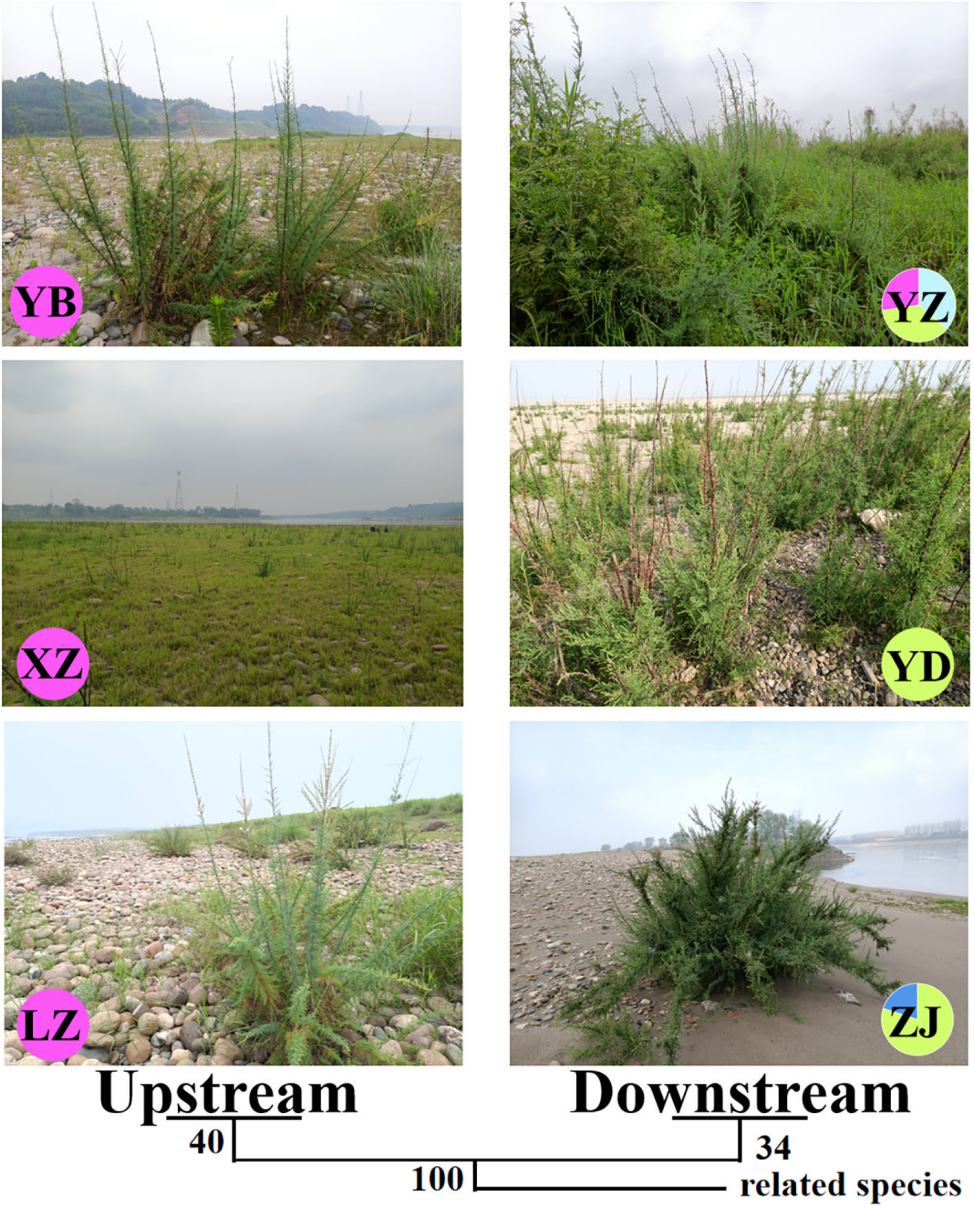
The habitats and phylogenetic tree of nuclear gene (ITS) in all *M. laxiflora* populations with related species. Each pie chart along the Yangzi River represents a population and each color represents a haplotype.

**Figure 3:**
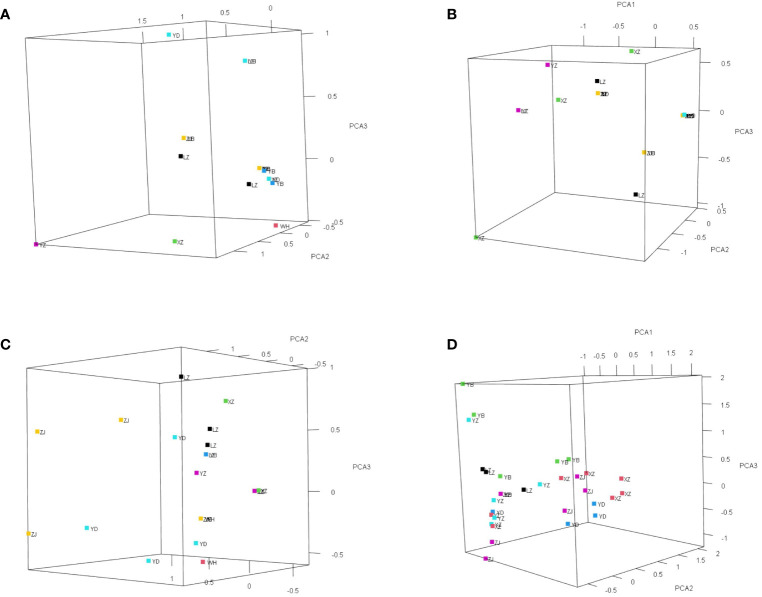
The resulting PCA plot of four chloroplast genes (*trnL-F*, *psbA-trnH*, *rps16*, and *rpl16*) for *M. laxiflora*. Each point in the plot represents a genetic sequence, and the colors indicate different groups obtained from a grouping file. **(A)** *trnL-F*. **(B)** *psbA-trnH*. **(C)** *rps16*. **(D)** *rpl16*.

One nuclear DNA (ITS) and four chloroplast fragments (*trnL-F*, *psbA-trnH*, *rps16*, and *rpl16*) were used to analyze 303 individuals from six natural populations of *Myricaria laxiflora*. The ITS and cpDNA haplotype frequencies of each population and the geographical distributions of haplotypes were shown in **Figure 1**. The total length of the aligned sequences of ITS was 696 bp containing four polymorphic sites (S) and two gene conversion tracts (474-538 in ZJ05 and ZJ06). The PCA plot showed that the ITS of all the natural populations clustered into four groups with the Yanzhi and Zhijiang populations (YZ and ZJ) clustered alone. The introduced individuals of Beijing (BJ) alone owned H1 (CGCA), and the other nuclear haplotypes (H2-H5) were shared by two groups upstream and downstream the Three Gorges Reservoir (TGR). The populations upstream the TGR were fixed for a single haplotype (H2: TGAT), and only one haplotype (H3: TGCA) was shared by the populations downstream the TGR. The resulting PCA and haplotypes were separated obviously, which indicates that the germplasm protection evaluation of nuclear gene was relatively complete in all wild populations with various plant types and habitats (**Figure 2**). The length of four sequenced cpDNA was 844 bp (*trnL-F*), 315 bp (*psbA-trnH*), 790 bp (*rps16*), and 1017 bp (*rpl16*), respectively. The conserved DNA regions within the four cpDNA were 76-317, 161-256, 187-347, 289-824, and 826-902, respectively. The total number of insertion–deletion events (InDels) analyzed, respectively, were 1, 3, 1, and 4 with non-significance of Tajima’s D in cpDNA. The S of cpDNA was 14, 10, 17, and 48 recovered with chloroplast haplotypes of 14 (T1-T14), 17 (P1-P17), 17 (R1-R17), and 33 (C1-C33), respectively (**Table 2**). Like nuclear haplotypes, a small number of cpDNA haplotypes (T2, P1 and P5, R2 and R5, C7) were shared between the upstream and downstream of the TGR (**Supplementary Figure 4**). Simultaneously, the phylogenetic tree showed that all haplotypes of four chloroplast fragments were mixed with each other, indicating that the haplotypes were not separated obviously. The results of PCA plot also provide same insights into the genetic variation and grouping patterns within the dataset in *M. laxiflora* populations (**Figure 3**).

**Table 2:** The polymorphism data and neutrality tests of nuclear gene (ITS) and chloroplast genes (*trnL-F*, *psbA-trnH*, *rps16*, and *rpl16*) for *M. laxiflora*.

Gene	Length (bp)	S	Eta	h	Hd	π	K	Tajima’s D	*P*	D*	*P*	F*	*P*	Fu’s Fs
ITS	696	4	4	5	0.602	0.00159	1.108	0.61138	*P* > 0.1	−0.13154	*P* > 0.1	0.11557	*P* > 0.1	0.296
*trnL-F*	844	14	15	14	0.598	0.00146	1.234	−1.86306	*P* < 0.05	−1.28064	*P* > 0.1	−1.75538	*P* > 0.1	−9.284
*psbA-trnH*	315	10	11	17	0.832	0.00694	2.165	−0.25291	*P* > 0.1	−0.34608	*P* > 0.1	−0.37196	*P* > 0.1	−8.304
*rps16*	790	17	17	17	0.801	0.0024	1.881	−1.6402	0.10 > *P* > 0.05	−2.44695	0.10 > *P* > 0.05	−2.57267	*P* < 0.05	−11.244
*rpl16*	1017	48	54	33	0.974	0.00812	8.229	−1.15785	*P* > 0.1	−1.01012	*P* > 0.10	−1.26683	*P* > 0.1	−17.378

D* means Fu and Li's D* test statistic; F* means Fu and Li's F* test statistic; the “*” means Significant variation.

The haplotype diversity (Hd) of ITS was 0.602, and nucleotide diversity (π) was 0.00159 among all populations. The Hd of cpDNA ranged from 0.598 to 0.974, and π ranged from 0.00146 to 0.00812 across four chloroplast fragments. The genetic mutations (Eta) and differences (K) of chloroplast genes was generally richer than that of nuclear gene. The haplotype diversity was generally high (Hd > 0.5), among which the nucleotide diversity of *psbA-trnH* and *rpl16* was high (π > 0.05), indicating that *M. laxiflora* has produced a large and stable population after a long period of evolution (**Table 2**).

### Population structure

**Figure 4:**
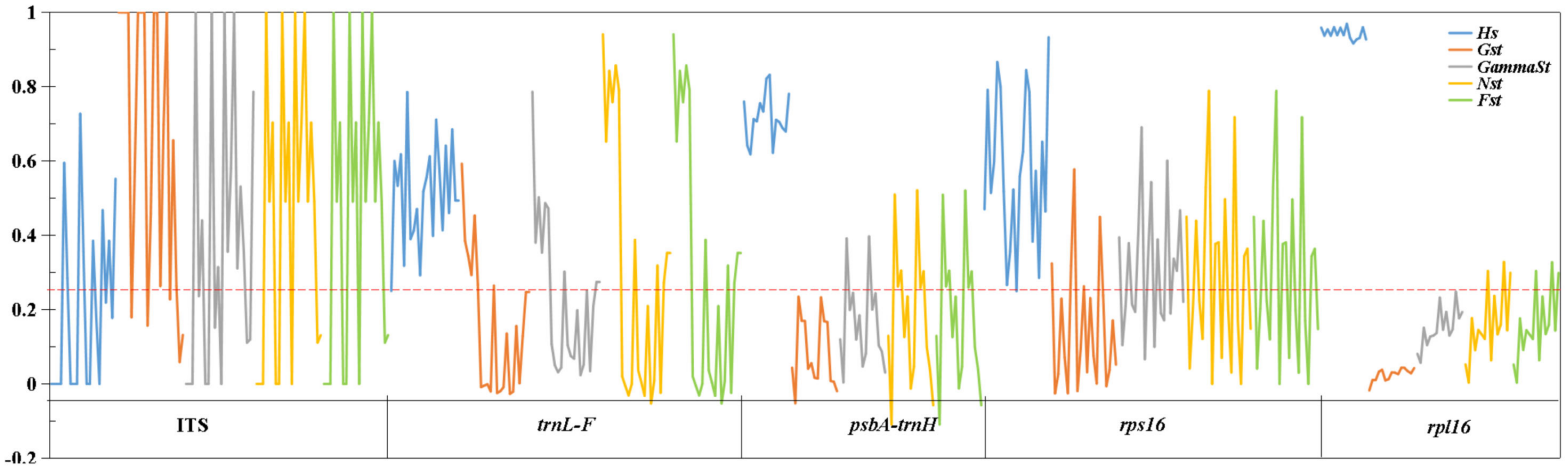
Genetic diversity and genetic differentiation of *M. laxiflora* based on nuclear gene (ITS) and chloroplast genes (*trnL-F*, *psbA-trnH*, *rps16*, and *rpl16*).

The generally high-genetic differentiation coefficient (Fst, Gst, and Nst > 0.25) was detected in the nuclear gene and chloroplast genes, indicating that there was a large genetic differentiation among the upstream and downstream populations. While the fixation index (Fst) of downstream populations was 0.05–0.15, there was a moderate proportion of genetic differentiation among populations. The Fst (1) between Yidu population (YD) and upstream populations in ITS showed that alleles were fixed and completely differentiated in Yidu population. The Fst (0) among the upstream populations in the nuclear gene showed that the population genetic structure was completely consistent, and there was no differentiation among the populations. The Fst (> 0.25) of chloroplast genes indicated that there was a great genetic differentiation among the upstream populations (**Figure 4**; **Supplementary Table 2**).

The AMOVA analysis performed at three hierarchical levels detected that the main genetic variation of *M. laxiflora* occurred among regions (59.34%) in ITS and within populations (64.86%–84.58%) in cpDNA (**Table 2**). The AMOVA of ITS indicated that distinct genetic differentiation patterns to those based on the cpDNA, that −7.24%–19.2% of overall variations was distributed among regions. The AMOVA indicated 15.12% of the total variation in the ITS populations could be accounted for among populations within regions, while 4.6%–41.7% in cpDNA. The estimate of differentiation among populations within regions (FST) ranges from 0.74 (ITS) to 0.15–0.35 (cpDNA), suggesting a large genetic differentiation among populations (**Table 3**).

**Table 3:** Analysis of molecular variance (AMOVA) of *M. laxiflora* based on nuclear gene (ITS) and chloroplast genes (*trnL-F*, *psbA-trnH*, *rps16*, and *rpl16*).

Genes	Source of variation	*df*	Sum of squares	Variance components	Percentage variation (%)	Fxation indices	
ITS	Among regions	1	14.446	0.48891	59.33892	FSC:	0.37185
	Among populations within regions	8	6.681	0.12458	15.11965	FST:	0.74459
	Within populations	47	9.891	0.21044	25.54143	FCT:	0.59339
*trnL-F*	Among regions	1	0.812	−0.04496	−7.24049	FSC:	0.26255
	Among populations within regions	5	9.194	0.17484	28.15547	FST:	0.20915
	Within populations	50	24.555	0.49111	79.08502	FCT:	−0.0724
*pabA-trnH*	Among regions	1	8.135	0.23117	19.2	FSC:	0.05692
	Among populations within regions	5	6.758	0.05539	4.6	FST:	0.23797
	Within populations	51	46.797	0.91759	76.2	FCT:	0.19198
*rps16*	Among regions	1	2.044	−0.06327	−6.56	FSC:	0.39134
	Among populations within regions	5	15.56	0.40225	41.7	FST:	0.35141
	Within populations	38	23.774	0.62563	64.86	FCT:	−0.0656
*rpl16*	Among regions	1	7.53	−0.08495	−2.03	FSC:	0.171
	Among populations within regions	4	35.83	0.73119	17.45	FST:	0.1542
	Within populations	40	141.792	3.5448	84.58	FCT:	−0.02027

FSC, differentiation among regions; FST, differentiation among populations within regions; FCT, differentiation within population.

**Figure 5:**
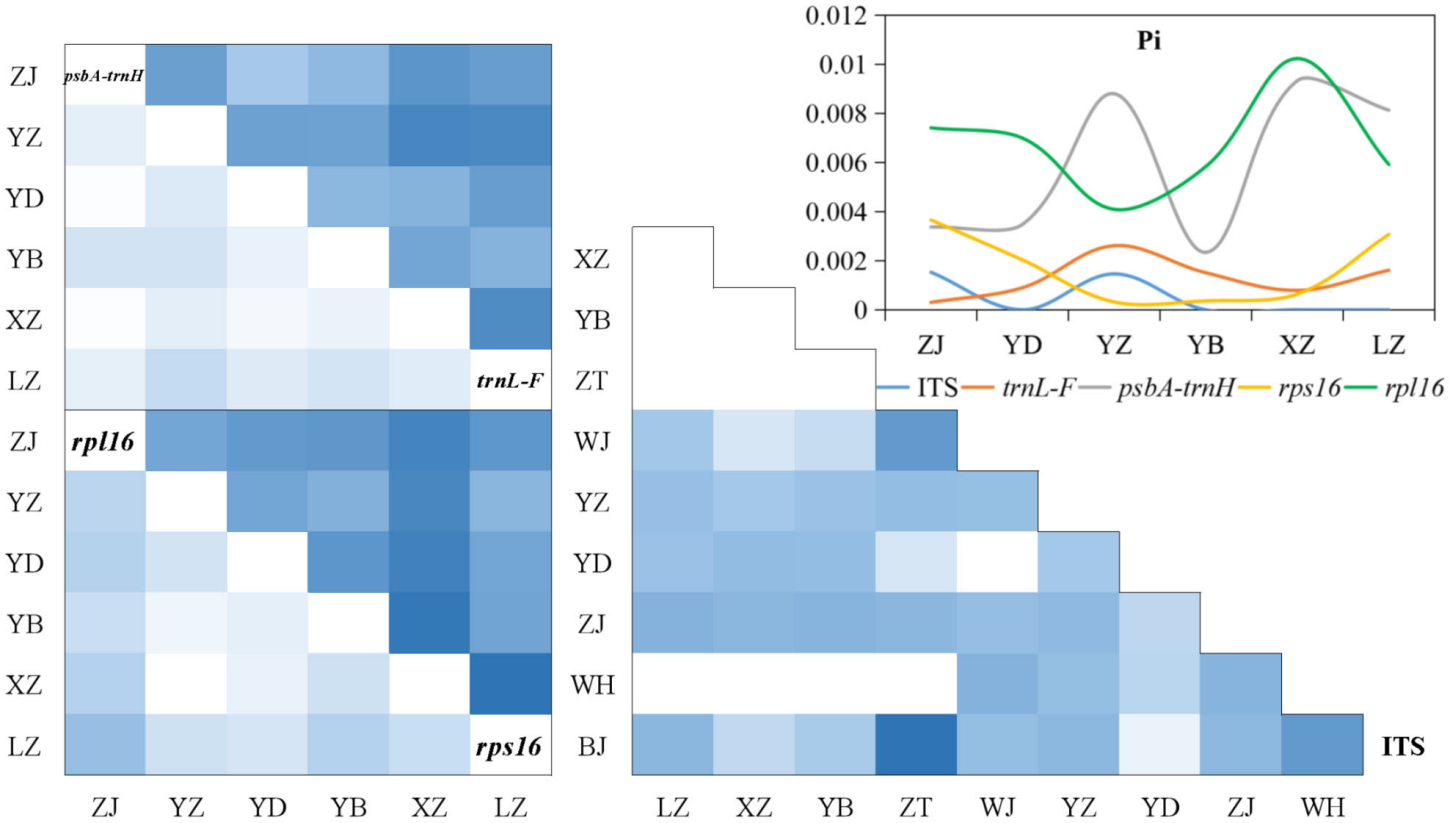
Population differences of *M. laxiflora* based on nuclear gene (ITS) and chloroplast genes (*trnL-F*, *psbA-trnH*, *rps16*, and *rpl16*).

**Figure 6:**
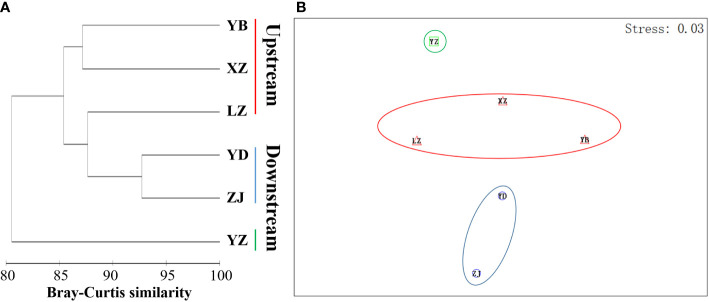
The MDS plot based on nucleotide diversity (Pi) of chloroplast genes (*trnL-F*, *psbA-trnH*, *rps16* and *rpl16*) in all *M. laxiflora* populations. **(A, B)** Dendrogram **(A)** and MDS plot **(B)** of upstream and downstream populations (Bray–Curtis similarity, standardization, group average; Stress = 0.03).

The analysis of population differences found that the genetic diversity of chloroplast genes within populations was significantly higher than that of nuclear gene, and the genetic diversity values (Pi) were high among the upstream and downstream regions of the TGR. The Pi (0.0015) of Zhijiang and Yanzhi populations (ZJ and YZ) was genetically differentiated contrast with others’ 0 in ITS. The population differences in cpDNA *psbA-trnH* and *rpl16* were relatively high compared to other genes. There was no population difference in the upstream populations of nuclear gene (ITS), and the Wuhan data (WH) from GenBank had no population difference from the upstream. However, there were significant differences from the downstream, indicating the Wuhan individuals of *M. laxiflora* analyzing in this paper might introduced from the upstream (**Figure 5**). The cluster analysis and the MDS plot based on nucleotide diversity (Pi) (**Supplementary Table 3**) of all chloroplast fragments revealed two clusters at the high Bray–Curtis similarity of 85%: (1) upstream populations and (2) downstream populations (**Figure 6**). The analysis showed one mismatched (YZ: **Figure 6B**) at the still high similarity of 81%, which did not affect the general pattern. The Stress = 0.03 < 0.05 indicates that the results are highly representative to measure the effectiveness of the MDS plot. The phylogenetic tree of nuclear gene (ITS) and four chloroplast fragments in all *M. laxiflora* populations have showed remnant populations have separated well to two groups which distributed upstream and downstream (**Figures 2**, **6**).

### Demographic history and distribution modeling

The demographic history of natural populations was determined by mismatch distribution and neutrality test based on ITS and cpDNA data. *P*-values (>0.05) of Tajima’s D and Fu’s Fs were non-significant for all genes in the neutrality test (**Table 2**). The mismatch distributions were multimodal and/or very ragged, which indicated populations were stable without rapid expansion recently (**Supplementary Figure 5**).

**Figure 7:**
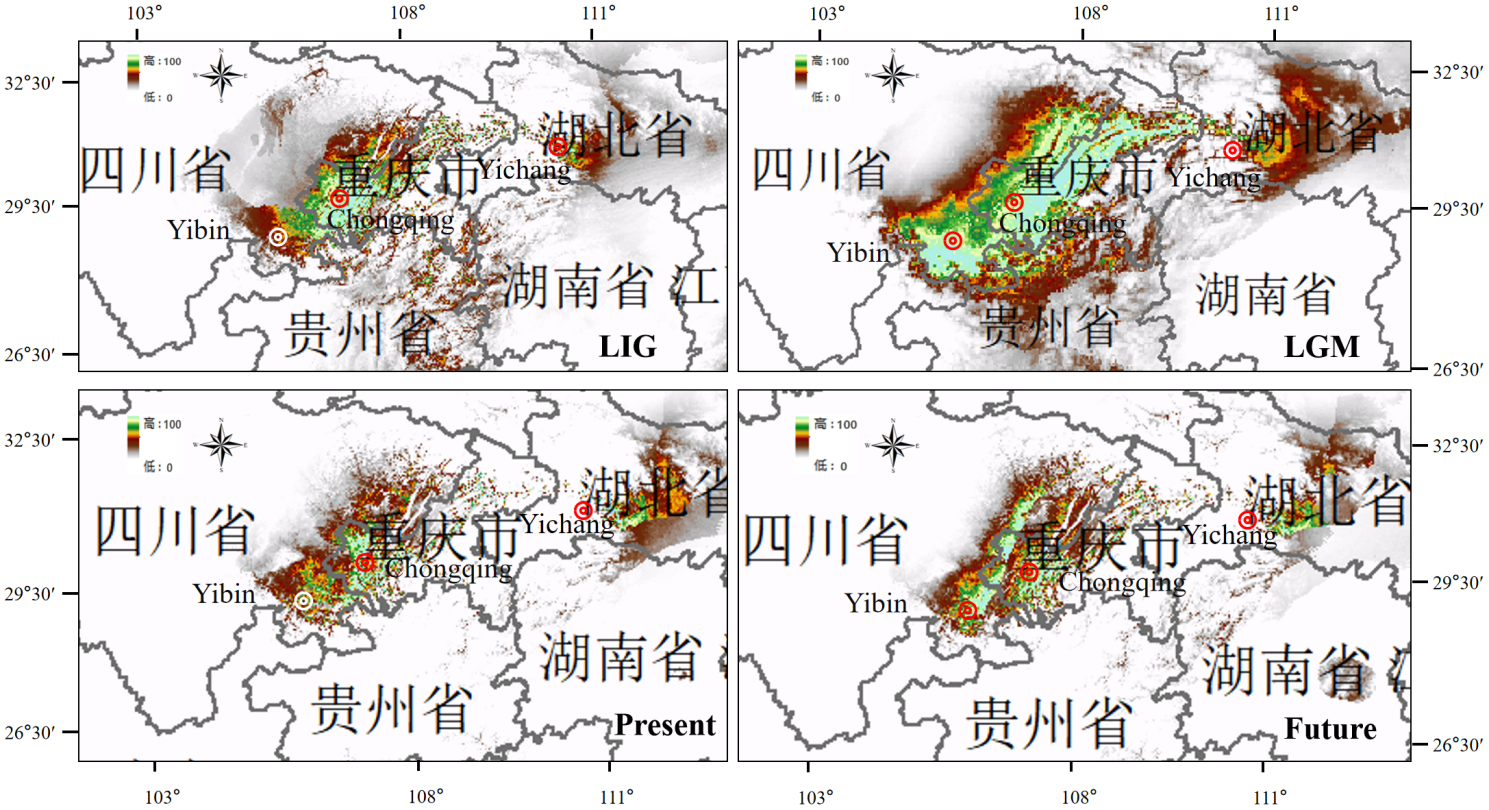
Potential distribution ranges of *M. laxiflora* during LIG, LGM, PresentF and future were simulated by using bioclimatic variables. Colors represent species climate adaptability.

The high AUC value (> 0.904) for the potentially suitable climate areas of *M. laxiflora* showed that the prediction results were extremely accurate and highly reliable. The distribution ranges predicted for *M. laxiflora* were consistent with the actual geographic distributions. The modeling results of six remnant populations and nine extirpated populations (**Table 1**) showed significant differences of the overall simulated distribution range in LIG, LGM, the present and the future. The main difference of *M. laxiflora* is focused on the change of the optimal distribution range (Green). The MaxEnt modeling results indicated the narrow scale distributions were originated in the Three Gorges area and showed doubled diffusion and migration from LIG to LGM as the climate decreased sharply. From LGM to the Present, the optimal distribution range was obviously reduced. The estimates of relative high contributions (>35%) of the environmental variables to the MaxEnt model was Precipitation of Wettest Quarter(Bio 6)and Precipitation of Driest Month (Bio 14) from LIG to the Present (**Supplementary Table 4**). The main factor contributing to the local habitat loss was due to the impoundment of the TGR, which has endangered all the natural populations of *M. laxiflora* there. The optimal distribution area in the future predicted that the distribution range of *M. laxiflora* might recover and expand steadily upstream and downstream of the TGR compared with that in the present (**Figure 7**).

## Discussion

### Population diversity and structure

The high population diversity caused by changeable environment is a result of the long evolutionary history for strong survival and adaptation of species (Soltis and Soltis, 1991; Avise and Hamrick, 1996). The molecular markers such as nuclear and chloroplast genes have been widely used to monitor the genetic diversity within and among populations of many endangered species, including *Adiantum nelumboides* (Wang et al., 2015) and *Davidia involucrata* (Chen et al., 2014). Non-similar to the chloroplast gene with uniparental maternal inheritance, the nuclear gene carries the genetic information of both parents, which can reflect the recent process of gene flow. In this study of genetic diversity, the analysis suggested that the six populations separated well to two groups which distributed upstream and downstream of the TGR. There were significant genetic differences in ITS and cpDNA haplotypes. The two gene conversion tracts of Zhijiang population (ZJ) in ITS were defined as the nonreciprocal transfer of information between homologous sequences for genome evolution (Santoyo et al., 2005). The H2 and H3 of nuclear gene could be used as identification of haplotypes for upstream and downstream population’s differentiation. The conserved DNA regions and InDels polymorphism within the four cpDNA could be caused by non-recombinant maternal inheritance of single parent. The Eta and K of cpDNA was generally richer than that of ITS with the high-haplotype diversity (Hd > 0.5) and nucleotide diversity (π > 0.05 in *psbA-trnH* and *rpl16* genes), owing to the breeding system of insect-mediated, selfing, and vegetative clonal reproduction and dispersal of wind and water (Wu et al., 1998; Wang et al., 2003; Liu et al., 2006). The high level of genetic diversity was also revealed by isozyme markers (Liu et al., 2010), ISSR (Tian et al., 2012), and AFLPs (Liu et al., 2006) within populations of *M. laxiflora*.

After a long period of evolution, a large and stable population was produced within *M. laxiflora*. However, the gene flow between the upstream and downstream populations was restricted by the TGR. The high-genetic differentiation coefficient (Fst, Gst, and Nst > 0.25), genetic variation (ITS: 59.34%) and population differences among regions indicated the seed/pollen dispersal capacity of *M. laxiflora* is very limited and the gene flow levels of populations are very low. Species with self-fertilization and asexual reproduction usually have a higher percentage of genetic differences among regions (Black-Samuelsson and Andersson, 1997; Gaudeul et al., 2000). Among the downstream populations, the coefficient of genetic differentiation revealed that a moderate level and detected alleles were fixed and completely differentiated in Yidu population, whereas the moderate Pi (0.0015) of Yanzhi and Zhijiang populations (YZ and ZJ) was also observed in ITS. An artifact of small-population size and reduced allelic diversity could be contributed to a moderate proportion among the downstream populations. Similar estimates of inter-population differentiation have also been observed in other riparian plants (Chen et al., 2006; Dong et al., 2007). The upstream populations were tested no genetic differentiation and population difference in the nuclear gene, but a large genetic differentiation in the chloroplast genes with many mutations occurred, which indicated that the long-term inundation of summer flooding and violent erosion of high-altitude glacial river from Hengduan Mountains have great impacts on the photosynthesis of endangered aquatic plants (Zheng et al., 2021). Similarly, the AMOVA showed that the genetic variation within the populations was large in cpDNA (64.86%–84.58%). The high Bray–Curtis similarity and highly representative to measure the effectiveness of the MDS plot have provided support for six populations separated to two groups, which distributed upstream and downstream. The mixed reproduction system with insect outcrossing pollination, selfing and vegetative clonal reproduction may be the main reasons to shape the present population structure of *M. laxiflora*. This mixed breeding system has probably played an important role in high inter-population genetic differentiation with an aggregate of linearly distributed populations (Hogbin and Peakall, 1999; Gaudeul et al., 2000; Prentis et al., 2004). Obviously, seed/pollen-mediated gene flow effective within each population diminishes logarithmically with increasing distance so as to form genetic isolation and limited gene flow among regions (Tero et al., 2003). The high divergences among the populations of *M. laxiflora* may be caused by drastic changes in the external environment and limited seed/pollen dispersal ability, which may be the results of long-term ecological adaptability of summer flooding stress.

### Population and range dynamics

Non-significant results of the neutrality test and multimodal distribution shapes of the mismatch distribution analysis (**Supplementary Figure 5**; **Table 3**) implied that the populations were stable without rapid expansion. According to our study mentioned above, six remnant populations of *M. laxiflora* have separated well to two groups which distributed upstream and downstream as the same of the MaxEnt modeling, which predicted that the simulated distribution range of *M. laxiflora* changed significantly during LIG, LGM, Present, and Future. The results of MaxEnt model showed that obvious unidirectional eastward migration via Yangtze River gorges watercourse-mediated from LIG to LGM. The initial habitat fragmentation could be triggered by the accumulation of local habitat loss of the impoundment of the TGR during the Present period (Liu et al., 2006, 2010) and might remain stable restoration with bidirectional diffusion in the Future.

The genetic structure and distribution of the surviving organisms can change dramatically through the drastic climate fluctuation generated by the recurrence of glaciation, which caused large-scale migration of organisms and natural disaster avoidance for organisms to survive (Taberlet et al., 1998). Regions relatively warm and humid for survival are often speculated as the glacial refuge of new species with high levels of genetic diversity (Tzedakis, 2002). The ancestral population of *M. laxiflora* originated in the eastern-Himalyas mountains during the Plio-Pleistocene, and the current distribution was caused by the uplift of the Yangtze River gorges (Wang et al., 2003, 2009; Zhang et al., 2014). The long evolutionary history, sexual and asexual reproduction, and habitat fragmentation may contribute to high-level genetic differentiation within *M. laxiflora* populations. The high levels of genetic diversity (Hd > 0.5) and endemic haplotypes were detected in populations, suggesting that the Three Gorges and its adjacent areas may have provided glacial refuges for *M. laxiflora* during the glaciation. The migration events of long distance dispersal from LIG to LGM were probably due to seeds dispersal or asexual propagules. The topography along the Yangtze River gorges isolated the entire natural range of *M. laxiflora* is complicated, and water flow should be the only driving force for unidirectional dispersal downstream. Steep cliffs and torrential water currents made seeds dispersal or vegetative propagules almost inaccessible to people and animal. The genetic diversity of the species did not reduced during the present, but an isolation-by-distance pattern of population structure probably developed under restricted gene flow (Hutchison and Templeton, 1999). The impoundment of the TGR made the upstream and downstream regions form new semi-flooded habitat for the growth of *M. laxiflora* with signs of stable restoration with bidirectional diffusion in the future. Based on the rescuing conservation of rare germplasm resources and the ecological restoration of water-level fluctuatation zones, the significant genetic differences of flood-tolerant plants have important theoretical and practical significance. This research is crucial to understand the high impact of environmental changes on the evolution of endangered rare plants (Stromberg, 2001).

### Enlightenment for rescuing conservation

The present study has important enlightenment for rescuing conservation of *M. laxiflora*, which is of great significance to ensure the diversity of genes and species. The loss of habitat due to environmental changes and other factors is the main factor contributing to the endangerment of *M. laxiflora* while not the loss of genetic diversity (Chen et al., 2005; Liu et al., 2006). The natural populations with high-genetic diversity have provided rich resources for rescuing conservation. The newly discovered habitat of the remnant populations are under threat of extirpation because of the seasonal flooding upstream and downstream of the TGR (Liu et al., 2006; Chen and Xie, 2007; Liu et al., 2010). The more effective measures on rescuing conservation should be taken to rapidly expand the number of populations, such as *ex-situ* conservation and artificial cultivation (Wang et al., 2003; Chen et al., 2005; Liu et al., 2006). Several satisfactory transplant sites have been established locally near the Three Gorges Dam by Yangtze River Rare Plant Research Institute with funding from the China Three Gorges Corporation. In addition, the stem with buds as explants of *M. laxiflora* has been successfully induced cluster buds under the research of this institute. Therefore, *ex-situ* conservation outside the TGR with low altitude provides a great reference for the new discovery of six remnant populations. In addition, the nature reserve upstream and downstream of the TGR should be urgently established to decrease human interference and increase the potential for *in-situ* conservation and reintroduction (Chen et al., 2005; Liu et al., 2010).

The main reproductive mode of *M. laxiflora* is seeds or vegetative propagules through unidirectional water transmission, which has restricted the gene flow and genetic drift in the long distance dispersal (Li et al., 2021). It is necessary to increase gene flow to prevent population degradation and maintain the maximum amount of genetic resources with appropriate strategies, which need to reduce inbreeding and artificially promote recruitment among the upstream and downstream populations. Furthermore, the moderate genetic differentiation of downstream populations and the presence of allele fixation in Yidu population (YD) indicate that the populations may have lost some extent alleles and genetic resources in the process of evolution and adaptation to the downstream environments (Wang et al., 2003; Liu et al., 2006). The completely distinct level of genetic differentiation among the upstream populations in the nuclear gene and chloroplast genes indicated artificial gene flow should be exercised before population genetic enhancement in case of outbreeding depression. Thus, appropriate measure combination of *ex-situ* and *in-situ* conservationist is significant important to maintain private alleles and population diversity.

This identified gene flow and range dynamics have provided support for the gene-flow and geology hypothesis that unidirectional demographical expansion of *M. laxiflora* from the upper palaeo-Chuan Jiang River area of Sichuan occurred sometime after the completion of the river channel through the TGR and the Yangtze River began to flow eastwards, which is also a climate refuge during climate oscillations in the Quaternary (Wu et al., 2003; Liu et al., 2006, 2009). The high contributions of Precipitation of Wettest Quarter and Driest Month from LIG to the Present have a significant correlation with seasonal fluctuations in water level (Guo et al., 2019; Xiong et al., 2019; Guo et al., 2023), which result in summer flooding stress for *M. laxiflora*. Long-term summer flooding has caused it to evolve the growth habit of summer dormancy before the onset of summer flooding to escape flooding stress (Chen et al., 2022). Thus, the hydrochory and climate dynamics of reversed seasonal fluctuations in water level and regulated summer flooding have high impacts on bidirectional adaptability and sustainability for *M. laxiflora* in new upstream and downstream habitats of the TGR. The reintroduction is one of the ways to realize the effective conservation, which is an important way to rebuild the wild population of *M. laxiflora* beyond simple *in-situ* and single species conservation. The reintroduction is also underway with substantial progress. Yanzhi population (YZ) was frequently used as experimental plot (Chen et al., 2019, 2022), which is also the largest remnant population of *M. laxiflor*a with various habitats (Huang et al., 2023), that are genetically differentiated firstly should be considered top priority to reintroduce individuals to the wild. As many different populations as possible should be saved to maximize genetic diversity and evolutionary potential to adapt to the new environment. Further integration of biotechnology, ecological technology, and engineering technology are necessary to achieve the restoration of reproduction, habitat, and inter-population. This study has provided valuable information for formulating conservation guidelines to maximize the chance of successful reintroduction of self-sustaining populations in the wild.

## Data availability statement

The datasets presented in this study can be found in online repositories. The names of the repository/repositories and accession number(s) can be found below: https://www.ncbi.nlm.nih.gov/genbank/, OK135371-OK135422, OK265112-OK265317, AY207486, EU240609 and EU240610, KJ808608 and KJ808609, KJ808639 and KJ808640, KJ808623 and KJ808624.

## Author contributions

HL: Data curation, Formal analysis, Investigation, Software, Validation, Visualization, Writing – original draft. GH: Conceptualization, Funding acquisition, Writing – review & editing. LQ: Project administration, Resources, Supervision, Writing – review & editing. JL: Methodology, Project administration, Writing – review & editing. YG: Investigation, Software, Writing – review & editing.

## Supplementary material

Supplementary Figure 1 The water level fluctuations upstream in Yibin and downstream in Yichang from 2014 to 2019.

Supplementary Figure 2 The PCR amplification confirmed by Gel electrophoresis analysis.

Supplementary Figure 3 The phylogenetic tree of nuclear gene (ITS) in all *M. laxiflora* populations with related species.

Supplementary Figure 4 Geographic distribution of four chloroplast genes (*trnL-F*, *psbA-trnH*, *rps16* and *rpl16*) haplotypes for *M. laxiflora* in all *M. laxiflora* populations. Each pie chart along the Yangzi River represents a population and each color represents a haplotype. Legends are arranged in increasing number of haplotypes.

Supplementary Figure 5 Mismatch distribution analysis of *M. laxiflora* based on nuclear gene (ITS) and chloroplast genes (*trnL-F*, *psbA-trnH*, *rps16* and *rpl16*). The line represents the distributions of an expected population expansion (Exp), the dashed line show observed (Obs) values.
