# Supplementary figures and images for: High effects of climate oscillations on population diversity and structure of endangered Myricaria laxiflora
Source: Front Plant Sci. 2024 Feb 28;15:1338711. doi: 10.3389/fpls.2024.1338711 (PMC10933041; doi:10.3389/fpls.2024.1338711)

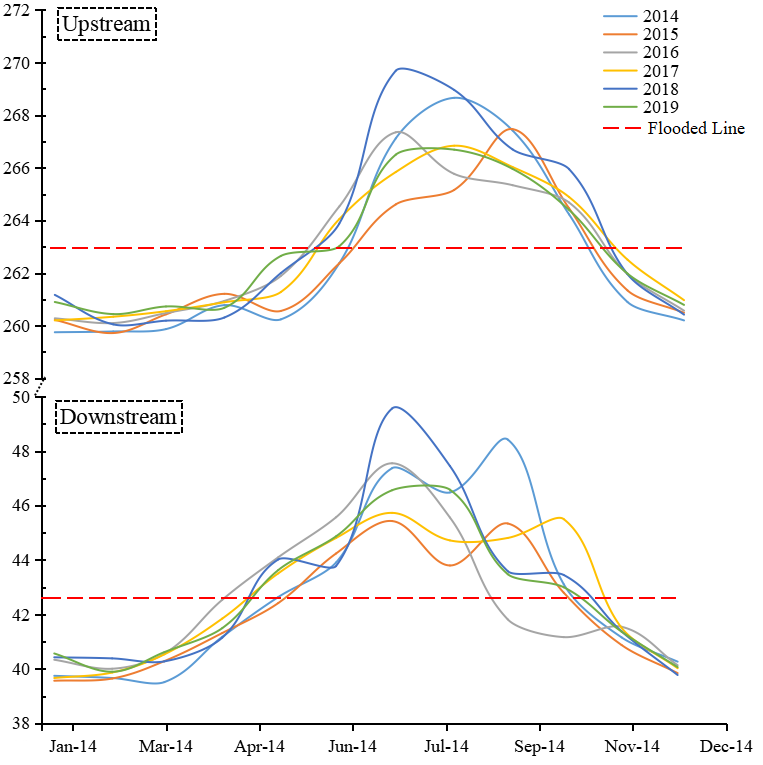

Supplement: Supplementary Figure 1 — The water level fluctuations upstream in Yibin and downstream in Yichang from 2014 to 2019. [file Image_1.tif]

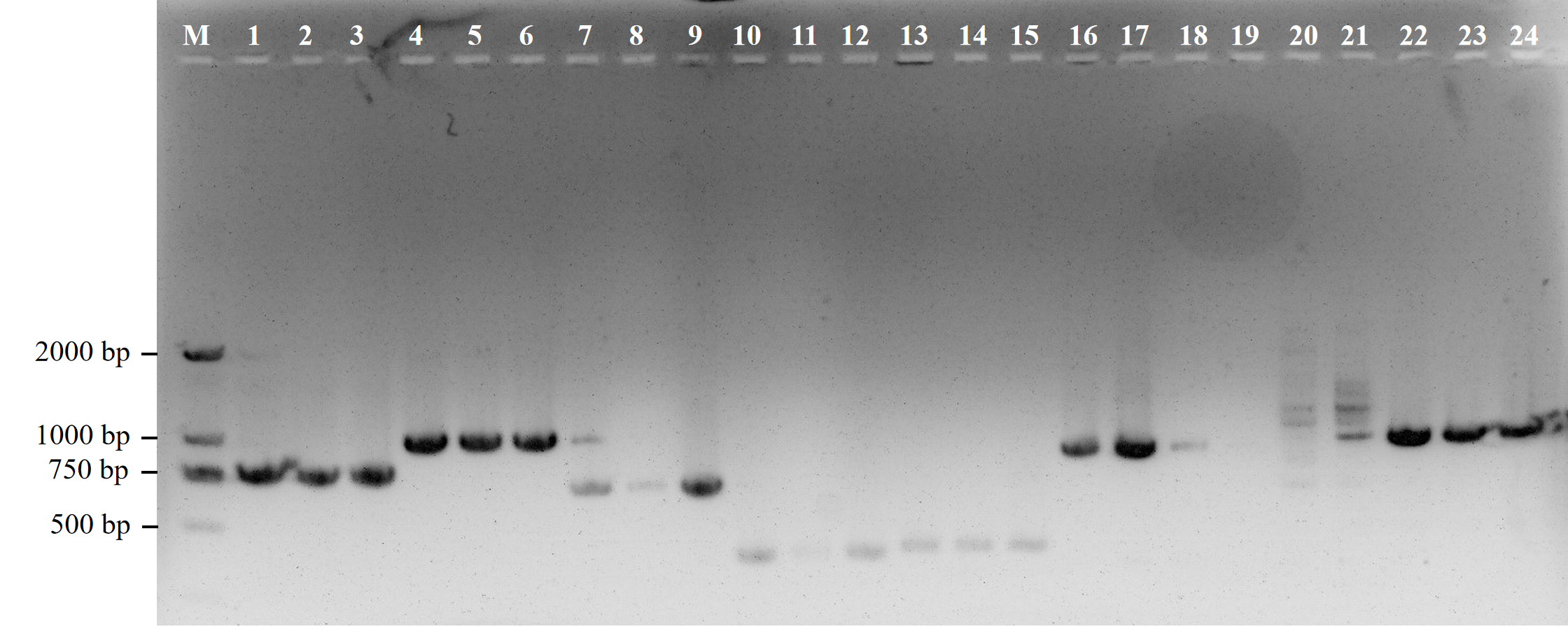

Supplement: Supplementary Figure 2 — The PCR amplification confirmed by Gel electrophoresis analysis. [file Image_2.tif]

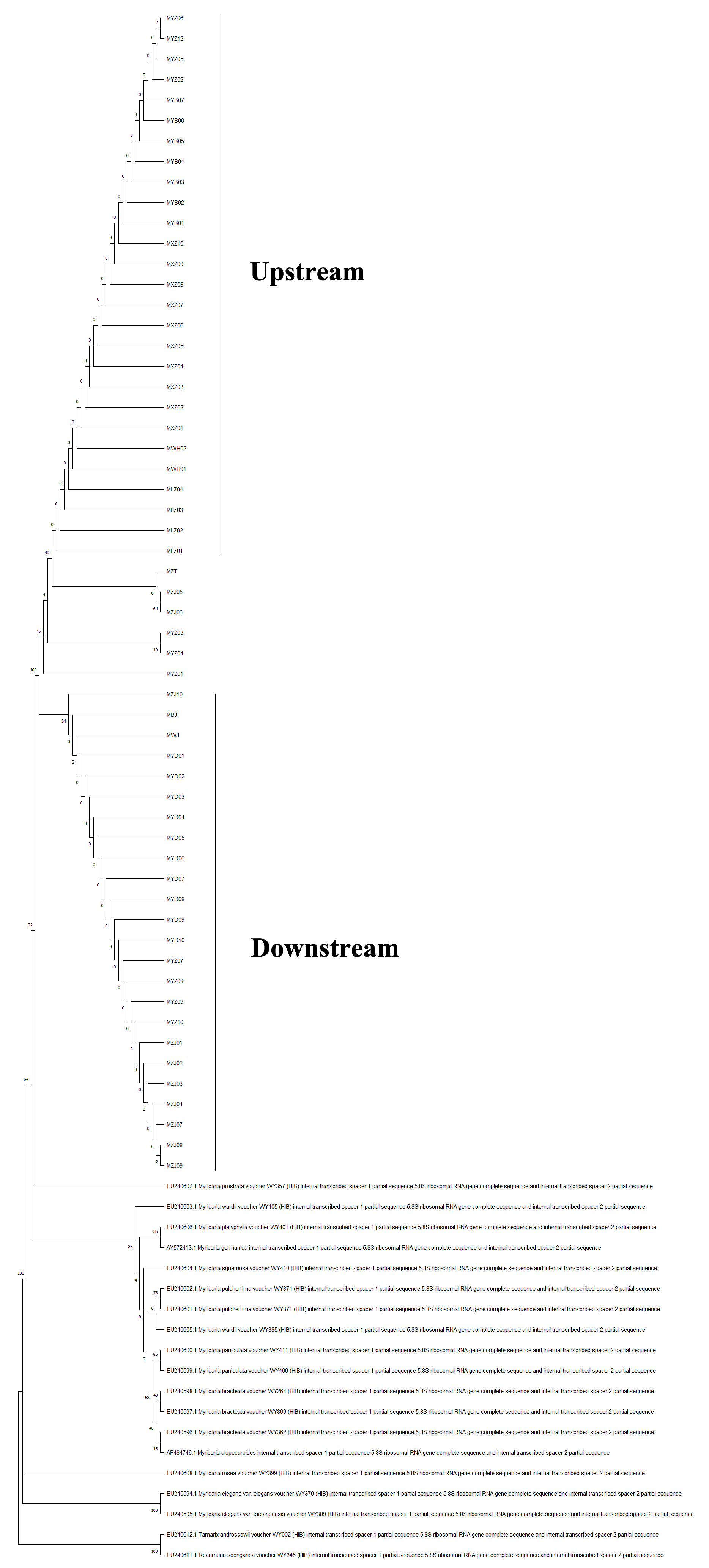

Supplement: Supplementary Figure 3 — The phylogenetic tree of nuclear gene (ITS) in all M. laxiflora populations with related species. [file Image_3.tif]

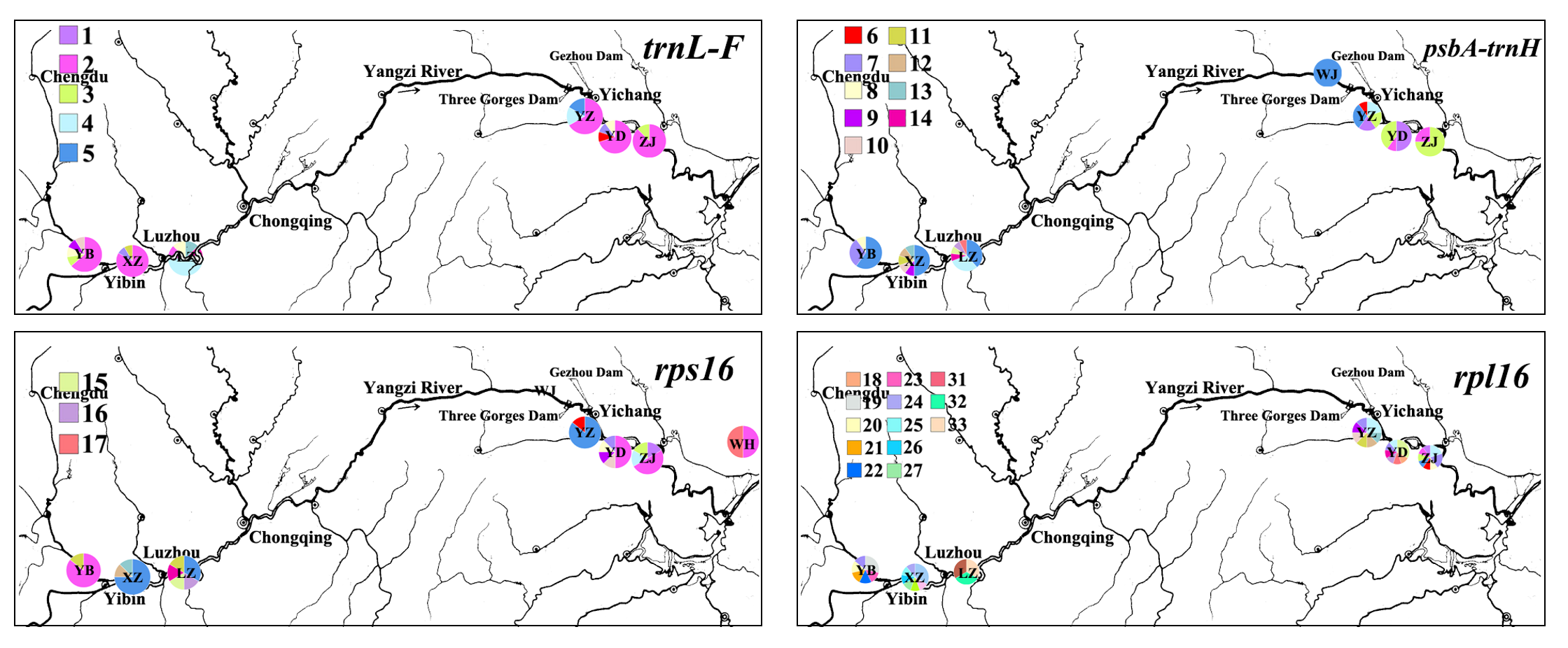

Supplement: Supplementary Figure 4 — Geographic distribution of four chloroplast genes (trnL-F, psbA-trnH, rps16 and rpl16) haplotypes for M. laxiflora in all M. laxiflora populations. Each pie chart along the Yangzi River represents a population and each color represents a haplotype. Legends are arranged in increasing number of haplotypes. [file Image_4.tif]

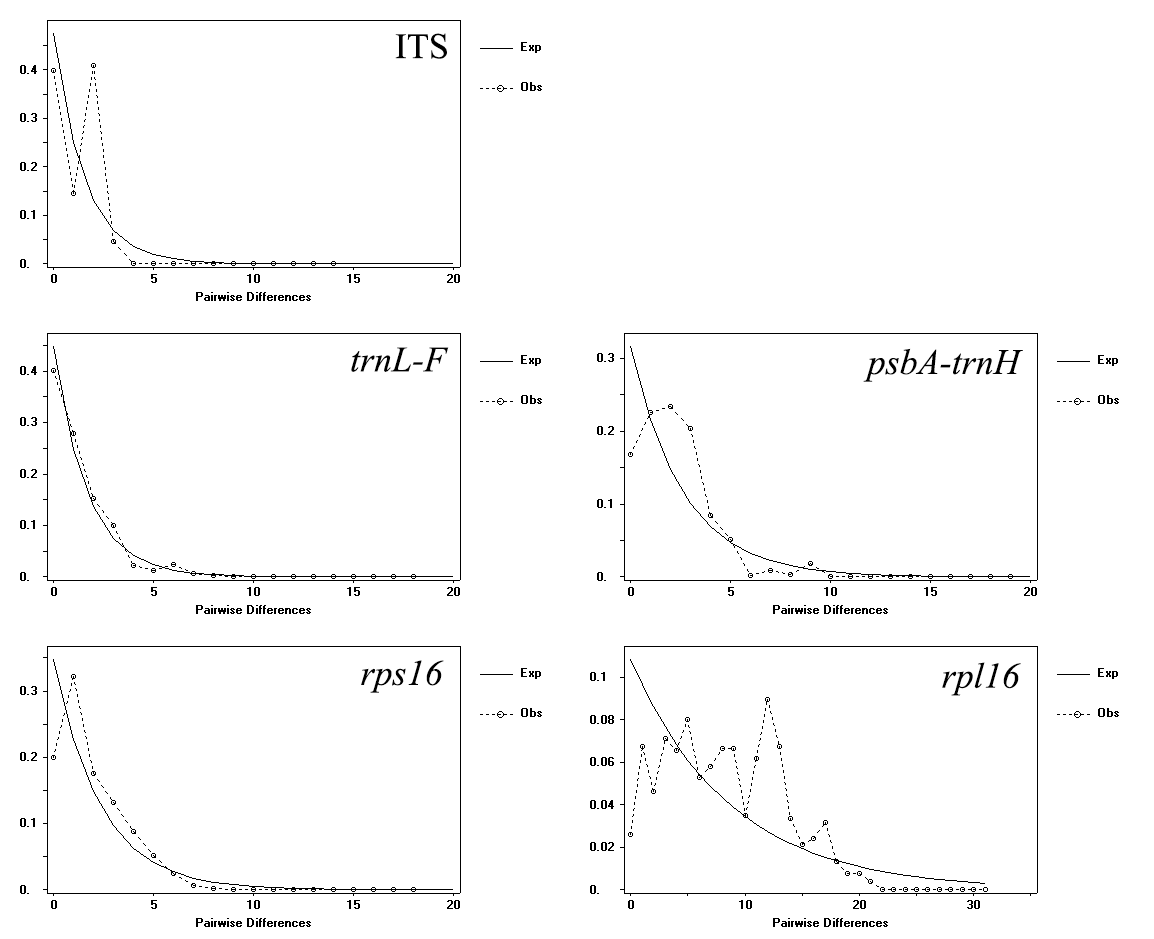

Supplement: Supplementary Figure 5 — Mismatch distribution analysis of M. laxiflora based on nuclear gene (ITS) and chloroplast genes (trnL-F, psbA-trnH, rps16 and rpl16). The line represents the distributions of an expected population expansion (Exp), the dashed line show observed (Obs) values. [file Image_5.tif]
